# Can the Tumor Deposits Be Counted as Metastatic Lymph Nodes in the UICC TNM Staging System for Colorectal Cancer?

**DOI:** 10.1371/journal.pone.0034087

**Published:** 2012-03-26

**Authors:** Yong-Xi Song, Peng Gao, Zhen-Ning Wang, Ji-Wang Liang, Zhe Sun, Mei-Xian Wang, Yu-Lan Dong, Xin-Fang Wang, Hui-Mian Xu

**Affiliations:** 1 Department of Surgical Oncology and General Surgery, First Hospital of China Medical University, Shenyang, People's Republic of China; 2 Department of Tumor Pathology and Surgical Oncology, First Hospital of China Medical University, Shenyang, People's Republic of China; Penn State Hershey Cancer Institute, United States of America

## Abstract

**Objective:**

The 7th edition of AJCC staging manual implicitly states that only T1 and T2 lesions that lack regional lymph node metastasis but have tumor deposit(s) will be classified in addition as N1c, though it is not consistent in that pN1c is also an option for pT3/T4a tumors in the staging table. Nevertheless, in this TNM classification, how to classify tumor deposits (TDs) in colorectal cancer patients with lymph node metastasis (LNM) and TDs simultaneously is still not clear. The aim of this study is to investigate the possibility of counting TDs as metastatic lymph nodes in TNM classification and to indentify its prognostic value for colorectal cancer patients.

**Methods and Results:**

In this retrospective study, 513 cases of colorectal cancer with LNM were reviewed. We proposed a novel pN (npN) category in which TDs were counted as metastatic lymph nodes in the TNM classification. Cancer-specific survival according to the npN or pN category was analyzed using Kaplan-Meier survival curves. Univariate and multivariate analyses were performed to indentify significant prognostic factors. Harrell's C statistic was used to test the predictive capacity of the prognostic models. The results revealed that the TD was a significant prognostic factor in colorectal cancer. Univariate and multivariate analyses uniformly indicated that the npN category was significantly correlated with prognosis. The results of Harrell's C statistical analysis demonstrated that the npN category exhibited a superior predictive capacity compared to the pN category of the 7th edition TNM classification. Moreover, we also found no significant prognostic differences in patients with or without TD in the same npN categories.

**Conclusions:**

The counting of TDs as metastatic lymph nodes in the TNM classification system is potentially superior to the classification in the 7th edition of the TNM staging system to assess prognosis and survival for colorectal cancer patients.

## Introduction

Colorectal cancer is the third most common malignancy in both men and women, and the second leading cause of cancer-related death in western developed countries [1], [2]. Currently, the tumor stage remains to be the most important determinant of prognosis in colorectal cancer and it is the basis for the authoritative patient management guidelines that influence most patient management decisions. The International Union Against Cancer (UICC)/American Joint Committee on Cancer (AJCC) TNM classification system is the principal staging systems utilized and therapeutic decisions are most often based on this classification system [3]. Great changes in the TNM staging system for colorectal cancer have occurred from the 5th edition to the 7th edition, particularly regarding the pN classification [4]–[6].

Tumor deposits (TDs) are defined as focal aggregates of adenocarcinoma located in the pericolic or perirectal fat discontinuous with the primary tumor [7]. Recently, several studies have reported on prognostic analyses of TDs in colorectal cancer, and found that the presence of TDs was an important prognostic factor [8]–[11]. Colorectal cancer patients with TDs exhibited a poorer prognosis and lower survival rate compared to patients without such lesions. Due to the significant value in clinical practice, TDs were still taken into account in the current 7th edition of TNM classification for colorectal cancer, and a new pN1c category was proposed. It states that only T1 and T2 lesions that lack regional lymph node metastasis but have tumor deposit(s) will be classified in addition as N1c, though it is not consistent in that pN1c is also an option for pT3/T4a tumors in the staging table [6]. Although the latest TNM classification states that the number of TDs should be recorded according to the categorization criteria, there are no guidelines on how to classify TDs in patients with lymph node metastasis(LNM) and TDs simultaneously. Therefore, this potentially impacts the accuracy of the classification, particularly how to assess these patients with colorectal cancer.

In the 7th edition of AJCC gastric cancer staging, pathologic assessment of the regional lymph nodes entails their removal and histologic examination to evaluate the total number of nodes, as well as the number that contain TDs, and TDs in the fat adjacent to a gastric regional LNM without evidence of residual lymph node tissue were considered as LNM [12]. In the light of these considerations, the aim of the present study is to investigate the possibility of counting TDs as metastatic lymph nodes in TNM classification when LNM and TDs exist simultaneously and to indentify its prognostic value for colorectal cancer patients.

## Methods

### Participants

Information on all patients with stage III colorectal cancer who underwent radical surgery at the Department of Surgical Oncology at the First Hospital of China Medical University from April 1994 to December 2007 were retrospectively collected, reviewed, and analyzed. Patients with any of the following criteria were excluded from the present study: (i) patients who died in the immediate postoperative period (within 30 days), (ii) patients with multiple adenocarcinomas of colon and rectum, (iii) patients with synchronous or metachronous tumors, (iv) patients who underwent neoadjuvant treatment, (v) patients who were classified as pN1c or had no LNM, (vi) patients with distant metastasis found preoperatively, (vii) patients with incomplete pathological data entries, and (viii) patients who were lost to follow-up. After considering the above criteria, there were 513 colorectal cancer patients in our study. The clinical data including age, gender, date of surgery, date of death (if applicable), cause of death, date of follow-up, location of the primary tumor, tumor size, histologic grade, venous invasion, lymphovascular invasion, depth of invasion, number of lymph nodes retrieved, number of LNM, and number of TDs were obtained. Tumors originating from cecum to sigmoid colon were defined as colon cancer, and tumors located in the rectum or rectosigmoid junction were considered as rectal cancer [13].

### Pathological procedures

Specimens were fixed in formalin and stained with hematoxylin and eosin(H&E). Sections were examined by two independent pathologists and confirmed by a third expert to make the final diagnosis. Disagreements regarding the diagnosis were resolved as a consensus upon re-review of the slides with all three pathologists [14].

### Classification methods

All patients were firstly classified according to the 7th edition of the TNM staging system. Then, we utilized a new method to reclassify all cases with colorectal cancer exhibiting TDs and LNM simultaneously, namely, we counted TDs as LNM in the novel pN category. For our study purpose, the novel pN category and the novel TNM staging system were recorded as npN category and nTNM staging system.

### Follow up

Postoperative follow-up was completed for the entire study population until November 2008. Median and mean follow-up periods were 30.72 months and 39.85±29.94 months (range: 1.1–164.3 months), respectively.

### Ethics statement

The study was approved by the Research Ethics Committee of China Medical University, China. Informed consent was obtained from all patients before participating in the study.

### Statistical Analysis

Continuous data were presented as the mean ± standard deviation(SD). Cancer-specific survival was analyzed using Kaplan-Meier survival curves, and comparisons were made using the log-rank test. Multivariate analysis was performed using Cox's proportional hazards model. The predictive power of the individual models was evaluated using Harrell's C statistic. A model with perfect predictive capacity (sensitivity and specificity of 100%) would have a Harrell's C statistic of 1.00 and the highest Harrell's C statistic was chosen as the best model [15], [16]. Statistical analyses and graphics were performed with PASW Statistics 18.0 software (SPSS, Inc., Somers, NY, USA) and STATA MP ver.10 (StataCorp LP, College Station, TX) statistical software. A value of P<0.05 was considered to be statistically significant.

## Results

Clinicopathological features of stage III colorectal cancer patients are listed in Table 1. Of the 513 patients, there were 277 (54.0%) males and 236 females (46.0%; ratio 1.2∶1) with a mean age of 59.63±11.78 years (median 61 years; range 20–85 years). Among these patients, 212 patients (41.3%) had colon cancer and 301 patients (58.7%) had rectum cancer. TDs were found in 151 of 513 patients (29.4%), and the mean number of TDs retrieved was 2.52±2.63 (range 1–17). The median and mean number of lymph nodes metastasis was 2 and 3.44±3.58 (range 1–28), respectively. The median and mean number of lymph nodes retrieved was 11 and 13.00±8.75 (range 1–81), respectively.

**Table 1:** Univariate analysis of the prognostic factors for 513 patients.

	n^a^	5-YSR^b^(%)	P
Gender			0.657
Male	277	46.0	
Female	236	47.4	
Age,year			0.112
≤60	252	49.6	
>60	261	43.6	
Tumor location			0.551
Colon	212	50.6	
Rectum	301	44.5	
Size			0.784
≤5.0 cm	313	46.2	
>5.0 cm	200	47.3	
Venous invasion			0.117
Positive	9	18.5	
Negative	504	47.5	
Histologic grade			0.021
Well	189	52.7	
Moderate	263	44.2	
Poor	61	35.1	
Lymphovascular invasion			<0.001
Positive	62	26.0	
Negative	451	49.5	
The presence or absence of TDs			<0.001
Positive	151	33.0	
Negative	362	52.9	
pT category			<0.001
pT1+pT2	65	60.0	
pT3	352	50.1	
pT4	96	29.8	
pN category			<0.001
pN1a	172	59.9	
pN1b	180	51.0	
pN2a	96	32.1	
pN2b	65	17.4	
npN^c^ category			<0.001
npN1a	138	60.7	
npN1b	167	56.6	
npN2a	115	32.9	
npN2b	93	22.0	
TNM staging system			<0.001
IIIa	56	66.2	
IIIb	362	50.8	
IIIc	95	20.9	
nTNM^d^ staging system			<0.001
IIIa	53	66.4	
IIIb	343	51.1	
IIIc	117	23.6	

**n^a^: Number of patients.**

**5-YSR^b^: 5-year accumulative survival rate.**

**npN^c^: novel pN category.**

**nTNM^d^: novel TNM staging system.**

**Figure 1:**
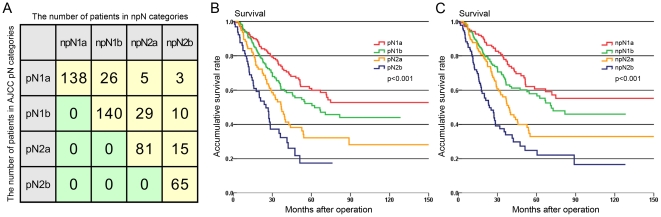
Stage migration in 88 patients and survival curves of patients according to the pN category and npN category. **A:** The number of patients who had stage migration in different subcategories. **B:** Survival curves showed different prognostic outcomes among patients with pN1a, pN1b, pN2a, and pN2b (P<0.001). **C:** Survival curves showed different prognostic outcomes among patients with npN1a, npN1b, npN2a, and npN2b (P<0.001).

Survival curves of 513 patients with colorectal cancer classified according to the different pN categories are shown in Fig. 1. As shown in Fig. 1A, upstaging occurred in 88 of 151 patients (58%) with TDs, and these patients staged by pN category showed a trend of migrating to a higher classification in nTNM staging system.There were significant prognostic differences among patients in the pN1a, pN1b, pN2a, and pN2b subcategories with regard to the pN category (Fig. 1B, P<0.001). Similarly, according to the npN category, significant differences were observed in the prognosis for these four subcategories (Fig. 1C, P<0.001). However, we found that certain patients showed stage migration as a result of the changes to the definition of the npN category.

Univariate analysis showed that the pN category, npN category, TNM classification and nTNM classification were significantly correlated with prognosis (P<0.001 for all) (Table 1). The variables pN and npN categories were highly correlated due to the fact that the npN category can be considered as an adjusted classification of the pN category. Therefore, multivariable models for the 513 patients were calculated separately for each variable to avoid bias in the estimation of variable effects (Table 2, 3). As shown in Tables 2 and 3, both the pN category and npN category were identified as independent prognostic factors by multivariate analyses (P<0.001 for both).

**Table 2:** Multivariate Analysis (Cox Proportional Hazard Model) of Prognostic Factors for 513 Patients with the pN category.

	HR^a^	95% CI^b^	P
pT category			<0.001
pT1+pT2*	1		
pT3	1.127	0.725–1.752	
pT4	2.229	1.380–3.598	
pN category			<0.001
pN1a*	1		
pN1b	1.265	0.898–1.783	
pN2a	1.676	1.148–2.445	
pN2b	2.817	1.858–4.272	
TDs	1.733	1.322–2.270	<0.001
Lymphovascular invasion	2.115	1.449–3.087	<0.001

**HR^a^: hazard ratio.**

**CI^b^: confidence interval.**

* **: reference category.**

**Table 3:** Multivariate Analysis (Cox Proportional Hazard Model) of Prognostic Factors for 513 Patients with the npN^a^ category.

	HR^a^	95% CI^b^	P
pT category			<0.001
T1+T2*	1		
T3	1.163	0.747–1.811	
T4	2.241	1.386–3.624	
nN category			<0.001
npN1a*	1		
npN1b	1.291	0.873–1.908	
npN2a	1.611	1.062–2.442	
npN2b	2.521	1.617–3.932	
TDs	1.399	1.037–1.887	0.028
Lymphovascular invasion	2.387	1.649–3.454	<0.001

**npN^a^: novel pN category.**

**HR^b^: hazard ratio.**

**CI^c^: confidence interval.**

* **: reference category.**

Next, the pN category, TNM classification, npN category and nTNM classification were measured by Harrell's C statistic to identify which has a superior predictive capacity. The results indicated that the npN category (Harrell's C = 0.6309; 95% CI:0.5939–0.6680) was superior to the pN category (Harrell's C = 0.6197; 95% CI:0.5821–0.6573). Additionally, between the TNM classification (Harrell's C = 0.6187; 95% CI:0.5868–0.6507) and the nTNM classification (Harrell's C = 0.6286; 95% CI:0.5960–0.6611), the latter was regarded as the perfect predictive parameter.

**Figure 2:**
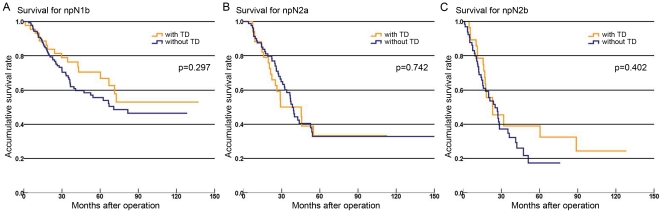
Survival curves of patients with or without TDs in the same npN categories. **A:** Survival curves showed similar prognostic outcomes between patients with or without TDs in npN1b (p = 0.297). **B:** Survival curves showed similar prognostic outcomes between patients with or without TDs in npN2a (p = 0.742). **C:** Survival curves showed similar prognostic outcomes between patients with or without TDs in npN2b (p = 0.402).

To study whether one TD carries the same weight as one positive lymph node in terms of patient prognosis, we focused on the patients with pure positive lymph nodes (without TDs) and compared the survival of patients with the number of TDs plus the number of LNM to patients with the same npN of pure positive lymph nodes only. The results showed no prognostic heterogeneity (Fig. 2, P>0.05).

## Discussion

Tumor deposits were first described by Gabriel *et al* in 1935, who concluded that these phenomena were the result of vascular tumor dissemination [17]. Since then, TDs in adjacent adipose tissue have become a well-known feature of colorectal cancer, and many studies have investigated the clinical significance of TDs in patients with colorectal cancer [8]–[10]. A meta-analysis of the survival of 3714 colorectal cancer patients confirmed the correlation of TDs with adverse prognosis, including increased local recurrence rates and increased development of distant metastasis [8]. Some authors have suggested that survival of the patients with such lesions is significantly lower compared to those without TDs [8], [11], [18]. Moreover, it has been reported that an increasing number and diameter of TDs are highly associated with an even worse clinical outcome [11], [18], [19]. Therefore, it is clear that TDs play an important role in the prognosis of colorectal cancer. In the present study, we examined the 5-year survival of patients according to clinical variables by univariate analysis. Not surprisingly, the presence of TDs was an important prognostic predictor in colorectal cancer. The postoperative 5-year survival of patients with and without TDs was 33% and 52.9%, respectively, and the former had a worse disease-free survival compared with TD-negative patients. We concluded that TDs could be potentially regarded as an adverse prognostic factor in colorectal cancer. This is consistent with previous reports that TDs exhibit a strong correlation with cancer aggressiveness [8], [11], [18].

The UICC/AJCC TNM staging system, although controversial, is considered as the most powerful and reliable predictor of prognosis for colorectal cancer patients around the world [20]. Over the past 13 years, this classification system for colorectal cancer has been revised three times. Whether TDs should be regarded as pT stage, pN stage, pM stage, or even be excluded from consideration in determining tumor stage also has changed several times. The 7th edition of AJCC staging manual implicitly states that only T1 and T2 lesions that lack regional lymph node metastasis but have tumor deposit(s) will be classified in addition as N1c, though it is not consistent in that pN1c is also an option for pT3/T4a tumors in the staging table. Tong *et al*, in a study of 1541 patients with colorectal cancer, suggested that the 7th edition of the TNM staging system on TDs satisfactorily predicted patients' outcome for those without LNM [21]. However, when colorectal cancer patients have LNM and TDs simultaneously, the TNM staging system dose not provide additional guidelines on staging for these patients. Therefore, the accuracy of the classification in these patients is potentially affected.

In the 7th edition of AJCC gastric cancer staging, TDs adjacent to a regional LNM without evidence of lymph node tissue were considered as LNM, and they were included in the number of lymph nodes for pathologic staging [12]. Wang *et al*, in their study of 1580 cases of gastric cancer, proposed that TDs could be classified based on their number and prognostic information should be incorporated into the TNM staging system [22]. In addition, TDs were generally considered to represent LNM in Japanese classification of colorectal carcinoma [23]. In the light of these considerations, we ventured to propose a new method in which TDs could be counted as LNM to subcategory pN stage in patients with colorectal cancer.

Univariate and multivariate analyses uniformly demonstrated that the pN category and npN category were significantly correlated with patient prognosis. Our results indicated that the npN category we proposed could satisfactorily predict the prognostic outcome of patients with colorectal cancer. However, we also found that stage migration occurred in some patients with various pN subcategories due to the change in defination. There was a noticeable trend in which patients with TDs in pN subcategories were upgraded to a higher stage under the nTNM classification. To determine whether the npN category and nTNM classification were superior to the pN category and TNM classification in terms of prediction capacity, we used Harrell's C statistic for data analyses, the results indicated that the npN category and nTNM classification exhibited a stronger predictive power compared to the other two models. Moreover, we also found no significant prognostic differences in patients with or without TDs in the same npN categories (Fig. 2). It suggested one TD carried the same weight as one positive lymph node in terms of patient prognosis. Therefore, we concluded that the npN category had superior clinical prognostic assessment in colorectal cancer patients.

The origin of TDs remains controversial until now. Some authors have proposed that TDs are derived from tumor growing inside or along lymphatic or vascular structures or nerves [8]. Others have suggested that TDs are potentially positive lymph nodes which are no longer recognizable because of their replacement by tumor cells [7]. Goldstein *et al*, in a study of 418 T3N+M0 patients with colorectal cancer, argued that the disease free survival impact of even small TDs was significant, suggesting that TDs of all sizes should be considered a single entity. The number and greatest dimension of TDs should be reported separately from lymph node metastasis [11]. In the present study, the results showed that the npN category and nTNM classification were superior to the pN category and TNM classification in assessing prognosis and survival of colorectal cancer patients. Taken together, it suggested that this new method could be used in the TNM staging system. According to our results, TDs had an adverse impact on prognosis and the influence of TDs on survival is potentially similar to LNM but different from hematogenous or implantation metastasis. We thought it was feasible that TDs was counted as LNM in the TNM classification of colorectal cancer. In addition, other authors have suggested that the perineural invasion associated with TDs was likely to occur in some colorectal cancer patients, resulting in a more adverse effect on 5-year survival [19], [24]. Thus, we should pay more attention to the TDs in clinical practice.

There are several limitations in this study. Our study is the result of a clinicopathological database of 513 Chinese colorectal cancer patients. Clearly, our conclusions showed the usual limitations of retrospective analysis from a single institution. At present, all studies on TDs in colorectal cancer are unicentric. On the other hand, although additional TD in the LNM confers a worse survival, the difference is rather small compared to using bona fide lymph node alone. Whether TD plus lymph node has any additional significant clinical impact to patient management need further investigation in larger samples. Therefore,we look forward to performing larger sample studies and international multicentric research on TDs in patients with colorectal cancer.

In conclusion, according to the results of our study, we found that it was feasible to count TDs as metastatic lymph nodes in the TNM staging system when assessing patients with colorectal cancer. The new method we proposed is potentially superior to the current 7th edition of TNM staging system for assessing prognosis and survival of colorectal cancer patients.
